# Impact of occupational environmental stressors on blood pressure changes and on incident cases of hypertension: a 5-year follow-up from the VISAT study

**DOI:** 10.1186/s12940-018-0423-9

**Published:** 2018-11-16

**Authors:** Samantha Huo Yung Kai, Jean-Bernard Ruidavets, Camille Carles, Jean-Claude Marquie, Vanina Bongard, Damien Leger, Jean Ferrieres, Yolande Esquirol

**Affiliations:** 1 UMR1027, INSERM, Université Paul Sabatier Toulouse III, 31000 Toulouse, France; 2 Univ. Bordeaux, INSERM UMR 1219, Equipe EPICENE, F33000 Bordeaux, France; 3 CHU de Bordeaux, Service de Médecine du Travail et pathologie professionnelle, F33000 Bordeaux, France; 4 CLLE, University of Toulouse, CNRS, UT2J, Toulouse, France; 5 Department of Epidemiology, CHU de Toulouse (Centre Hospitalier Universitaire), 31062 Toulouse, France; 6 Sorbonne Paris Cité, APHP, Hôtel Dieu, Centre du Sommeil et de la Vigilance et EA 7330 VIFASOM, Université Paris Descartes, 75004 Paris, France; 7 Department of Cardiology, CHU de Toulouse (Centre Hospitalier Universitaire), 31062 Toulouse, France; 8 Occupational Health department, CHU-Toulouse, 31000 Toulouse, France; 9 Faculté de médecine, 37 allées jules Guesde, 31000 Toulouse, France

**Keywords:** Occupational environmental stressors, Occupational exposure, Blood pressure, Hypertension

## Abstract

**Background:**

The role of occupational stressors (OS) on blood pressure (BP) is often suspected, but asserting its impact remains uncertain. Our goal was to evaluate their impact on BP increase and on incident cases of hypertension over a 5-year period.

**Methods:**

One thousand, one hundred and fifty-six men and women from the French prospective VISAT study were followed up over five-years (T1 to T2). Exposures to a large panel of OS (physical, organizational, psychosocial and employment categories) were collected. Linear and logistic regressions were used to assess associations between OS and T2-T1 SBP difference and incident cases of hypertension. They were performed to determine the role of OS first considered separately, then in combination, in crude and adjusted models for main cardiovascular risk factors (gender, age, education, BMI, lifestyle habits and medical history).

**Results:**

For initial SBP level < 130 mmHg, carrying loads, intense noise exposure, working more than 48 h/week, active and high strain tended to be associated with an SBP difference increase, while job recognition was associated with a decrease. After adjustment, only significant associations with job strain and job recognition persisted. For initial SBP level ≥ 130 mmHg, being exposed to an active job strain was positively associated with T2-T1 SBP difference only in unadjusted model. Considering all the OS, the recognition of completed tasks had a major protective role. No impact of OS on incident cases of hypertension was observed.

**Conclusion:**

Associations between OS and SBP were observed mainly when initial SBP is within the normal range, and are mainly explained by cardiovascular factors, requiring physician’s particular attention to people exposed to these OS. VISAT study is registered in “LE PORTAIL EPIDEMIOLOGIE – FRANCE- AVIESAN –ID 3666”.

**Electronic supplementary material:**

The online version of this article (10.1186/s12940-018-0423-9) contains supplementary material, which is available to authorized users.

## Background

For the past decades, research has increasingly been focusing on the impact of occupational environmental stressors (OS) (also named occupational risks) on health, highlighting their potential role in the development of prevention strategies and in the promotion of public health policy. Hypertension (HBP) is a major risk for cardiovascular diseases (CVD) [1] and has been confirmed in guidelines as the major objective of cardiovascular disease prevention [2]. Recent studies have provided greater understanding of the consequences of some constraints on outcomes related to CVD and risk factors [3–6].

Concerning blood pressure (BP), the impact of OS has been little investigated. For most of them, the conclusion remains unclear. Regarding psychological factors, demand–control–support and effort–reward imbalance models have been frequently used [7]. Few prospective studies were carried out, but most of them confirmed an adverse association between job strain and mean BP level, without consistent results regarding hypertension occurrence. Regarding organizational stressors, shift work was one of the most investigated with a suspected higher risk of hypertension among shift workers compared to day workers [8]. Concerning occupational physical stressors such as the exposure to intense noise or carrying heavy loads, a specific effect on BP has not been well established yet [9–11].

The heterogeneous results can be potentially explained by: i) the inconstant adjustments for the ‘traditional’ cardiovascular risk factors, and ii) not considering the simultaneous exposition to different OS [12]. Moreover, the initial level of BP which can interact with the effect of OS on BP changes should be considered [11, 13]. Thus, it is difficult to conclude on the effect of OS on BP.

The VISAT (ageing, health and work) study is one of the first to explore the impact of OS in a long-term investigation including BP change and hypertension. The objective was to assess the impact of different types of OS on changes in arterial SBP and on incident cases of hypertension during a 5-year follow-up with analyses adjusted for the main individual cardiovascular risk factors (gender, age, education, Body Mass Index, lifestyle habits and medical history). First, each OS was studied separately and their combined impact was assessed.

## Methods

### Design and population study

The French prospective VISAT study (VIeillissement SAnté Travail), designed to analyse the relationships between age, health and work conditions (as available elsewhere [14]) was used to analyse data from two collections with a 5-year interval (2001, T1 and 2006, T2). This study included participants born in 1934, 1944, 1954 or 1964 at T0. Data about 2284 participants were collected through self–administered questionnaires, medical questionnaires and clinical examinations conducted by trained occupational physicians during the mandatory and periodic health visits. Among participants, 55.0% (1257) were followed at T2 and data on SBP and hypertension were available for 1156 people. Comparisons of individual characteristics between included and drop-out participants are available in Additional file 1 (drop-outs were older, had a lower education level, were more likely to have history of diabetes and hypercholesterolemia, and to be treated for HBP at T1).

Participants were informed and volunteered to participate in the VISAT study. Informed consent was sought and granted. The VISAT project obtained agreement from the French National Committee (CNIL) and from an institutional ethic committee in accordance with the French law. All the procedures are in accordance with the declaration of Helsinki in human research.

### Blood pressure measurement

At each health visit, brachial BP was measured non-invasively three times (with a one-minute interval at least), in the presence of the practitioner, using an automatic standard sphygmomanometer (OMRON 705CP) with adapted arm cuff. Measurement was performed in a back-supported seated position, the arm placed at the same level as the heart, with a 5-min rest at least and without eating, smoking, and exercising at least 30 min before medical examination. The mean of the three measures was used for data analysis (see Additional file 2).

The within-visit SBP and DBP variability was evaluated and expressed as standard deviation (SD): concerning SBP between and within-individuals, SDs were respectively 18.6 and 6.6 mmHg at T1, and 18.8 and 5.5 mmHg at T2; for DBP SDs were respectively 11.6 and 5.8 mmHg at T1, and 11.2 and 4.4 mmHg at T2.

Treatment for hypertension was recorded at T1 and T2 and dichotomized into two categories (yes/no). Because SBP is currently considered as a better predictive risk factor than DBP regarding cardiovascular risk (2013 ESH/ESC Guidelines), broadly used in nomograms predicting CVD risk (Framingham score) or cardiovascular death (ESC SCORE) and in order to allow comparisons with prior publications examining the impact of OS on BP [9, 11] and given the modifications of DBP with age and stressors [15], the analyses in the current study were focused on SBP rather than on DBP.

### Assessment of individual characteristics

Gender (male/female), age categories (32, 42, 52 and 62 years old), body mass index (BMI) expressed in kg/m^2^ (mean(SD) and BMI < 25 or ≥ 25 kg/m^2^), smoking habits (current-former/never smoker), leisure time physical activity (PA) (none-slightly active/active-very active, corresponding to participants exercising intensely at least 20 min, twice a week), education attainment (≤A-degree level/>A-degree level), history of diabetes (yes/no) and history of hypercholesterolemia (yes/no) were collected. Daily alcohol intake was evaluated from the question ‘do you drink every day?’ (No/Yes, but able to stop for 4 days/Yes and unable to stop for 4 days). The working status (currently employed/retired) at T1 and at T2, and the change of job between T1 and T2 were collected.

### Assessment of occupational exposure

The questions used were similar to those used in the European survey on working conditions (http://www.eurofound.europa.eu/surveys/ewcs/2010/documents/technical.pdf, Gallup Europe 5th European Working Conditions Survey, 2010. Technical Report. 2010), in the ESTEV study [16, 17] and in the previous VISAT articles [18, 19].

OS were divided into 4 categories. Each constraint was dichotomized: exposed at baseline or in the preceding five years/never exposed during the working periods.

#### Physical constraints

Carrying heavy objects or intense physical activity; intense noise exposure (cannot hear a person who is 2–3 m away even if talking loudly).

#### Organisational constraints

Working at weekends; often working more than 48 h/week; rotating shift work; bedtime after midnight or getting up before 5 AM because of working hours and commuting time.

#### Psychosocial constraints

Repetitive tasks under time pressure, recognition of completed tasks and jobs with productivity-related income.

Using the method applied in the ESTEV [17] and VISAT studies [18] the decision latitude (DL) and the psychological demand (PD) were analysed using a proxy of the Karasek model [20]. By combining the levels (high or low-median) of each dimension, four different job strain categories were defined: ‘low job strain’ (low PD and high DL); ‘passive job’ (low PD and low DL); ‘active work’ (high PD and high DL); ‘high job strain’ (high PD and low DL).

#### Assessment of employment-related factors

Tertiles of age at first job (< 18/18–20 (reference)/> 20 years old) and the socio-professional status (white/blue collars).

### Statistical methods

In baseline descriptive analyses, the percentage for categorical variables, the mean and SD for continuous variables were provided. Change in BP was computed as the difference between BP measured at T2 and at T1.

SBP change was first defined in the whole sample, then according to the baseline SBP level, SBP < 130 mmHg and SBP ≥ 130 mmHg, defined as the cut-off of high normal BP by 2013 ESC guidelines [2]. Hypertension was defined with a SBP ≥ 140 mmHg and/or a DBP ≥ 90 mmHg and/or taking a treatment for hypertension. Participants without hypertension at T1 and with hypertension at T2 were used to analyse the association between OS and hypertension incident cases during the 5-year follow-up.

Firstly, bivariate analyses between the SBP difference or hypertension incident cases and the potentially explanatory variables (individual and occupational) were assessed using Chi^2^, Fisher’s exact test and Student or Mann-Whitney tests.

Incidence of hypertension was expressed in percentage for 1000 person-year.

Secondly, while individual and OS of SBP changes were assessed using linear regression models, hypertension incident cases were studied through a logistic regression analysis. In these regressions, OS were analysed separately and considered as the main explanatory variables. All models were systematically adjusted for the following individual variables measured at baseline: age group at eligibility, gender, BMI, smoking habits, daily alcohol intake, leisure time PA, history of diabetes, history of hypercholesterolemia, treatment for hypertension at T1 and T2 (adjustment not performed when assessing determinants of hypertension incident cases), educational attainment, working status (at T1 and T2), and initial BP level (for the whole population). The absence of collinearity between the explanatory variables was checked in the models. Odds ratios (OR), 95% confidence interval (CI), β linear coefficients, standard errors and *p*-values were computed.

Thirdly, in backward logistic and linear regressions, OS were considered jointly as explanatory variables to investigate the potential role of combined occupational explanatory factors on the T2-T1 SBP difference and on incident cases of hypertension over the 5-year follow-up, after adjustments for individual factors. The determinants which were associated with the dependent variable in bivariate analyses with a p-value< 0.20 were included in the a priori models. Then, explanatory variables were removed from the model one by one after likelihood ratio tests (considered significant if *p* < 0.05) to compare nested models. No interactions between OS and the confounders were observed in final models.

Significance level was set at 0.05. Statistical analyses were performed using Stata (StataCorp, 2013. Stata Statistical Software: Release 13. College Station, TX: StataCorp LP).

## Results

Mean T2-T1 differences were respectively evaluated to 1.5 mmHg (SD = 14.9) for SBP, and − 0.8 mmHg (SD = 10.7) for DBP, in the whole sample. Fifty-eight percent of the participants had an initial SBP < 130 mmHg. The mean SBP difference between T2 and T1 was 5.5 mmHg (SD = 12.9) and − 4.1 mmHg (SD = 15.8) respectively when initial SBP was < 130 mmHg and ≥ 130 mmHg. Overall participants with initial SBP level ≥ 130 mmHg were more exposed to OS (Additional file 3). Concerning hypertension, 33.0% (381) prevalent cases were inventoried at T1 (36.6% of them were treated at T2); 134 (17.3%) incident cases of hypertension occurred between T1 and T2 among people without hypertension at T1 (15.7% of them were treated for hypertension at T2). Incidence of hypertension among the studied sample was 34.7 [95% CI: 29.3; 41.1] per 1000 person-year.

As expected, the main cardiovascular risk factors were more frequently observed among participants who developed hypertension between visits (Table 1).

**Table 1:** Individual characteristics according to SBP difference and incidence of hypertension: 5 year-follow-up

	Whole sample (*N* = 1156)			Incident cases of hypertension at T2 Participants without hypertension at T1			
	%	Difference T2-T1		HBP incidence (*N* = 775)	No HBP (*N* = 641)	HBP (*N* = 134)	
		Mean ± SD	*p*	/1000 person-year	%	%	*p*
Age group at eligibility							
32 y	31.3	0.88 ± 13.33	0.359	19.4	43.7	22.4	**< 0.001**
42 y	33.3	1.79 ± 13.96		36.0	36.5	38.1	
52–62 y	35.4	1.83 ± 16.97		59.2	19.8	39.6	
Gender							
Male	52.7	0.54 ± 15.37	**0.018**	46.9	40.4	58.2	**< 0.001**
Female	47.3	2.61 ± 14.31		25.5	59.6	41.8	
BMI at T1							
< 25 kg/m^2^	52.8	1.6 ± 13.67	0.519	23.6	67.9	43.8	**< 0.001**
≥ 25 kg/m^2^	47.2	1.17 ± 16.05		53.4	32.1	56.2	
Smoking at T1							
No	71.7	0.96 ± 15.01	**0.067**	30.4	71.6	64.2	
Yes	28.3	2.78 ± 14.48		40.1	28.4	35.8	0.101
Daily alcohol intake at T1							
No	71.5	2.39 ± 14.46	**0.006**	30.0	82.1	70.2	**0.008**
Yes, able to stop	23.5	−0.37 ± 15.49		52.1	15.6	26.0	
Yes, unable to stop	5.0	−2.94 ± 16.78		51.0	2.4	3.8	
Leisure physical activity at T1							
None / Slightly active	57.4	1.07 ± 15.22	0.260	35.2	55.1	57.4	0.637
Active / Very active	42.6	2.08 ± 14.46		32.9	44.9	42.6	
Educational attainment							
≤ High school degree	67.7	1.36 ± 15.46	0.633	39.4	60.5	70.1	**0.036**
> High school degree	32.3	1.87 ± 13.7		27.3	39.5	29.9	
Diabetic at T1							
No	97.8	1.43 ± 14.87	0.532	34.3	99.2	97.8	0.146
Yes	2.2	5.1 ± 16.18		71.3	0.8	2.2	
Hypercholesterolemia at T1							
No	84.9	1.52 ± 14.77	0.883	32.8	88.9	82.8	**0.050**
Yes	15.1	1.34 ± 15.61		48.9	11.1	17.2	
Initial SBP level							
< 130 mmHg	58.2	5.5 ± 12.9	**< 0.001**	24.6	86.0	57.5	**< 0.001**
≥ 130 mmHg	41.8	−4.1 ± 15.8		78.7	14.0	42.5	

*Footnotes*: percentages, means (SD) or medians and interquartile ranges. Categorical variables were assessed using Chi-squared or Fisher’s exact test, continuous variables were assessed using Student’s t test or Mann-Whitney test

Significance of *p*-value < 0.5 are set in bold

The results from bivariate analyses between OS at T1 and i) SBP difference over 5 years in the whole sample and when considering the initial level of SBP ii) incident cases of hypertension, are provided in Additional file 4. Although a job change was recorded among 9.2% of the participants, it did not interact with BP change (all sample: *p* = 0.57 for SBP, *p* = 0.99 for DBP) and with hypertension cases (*p* = 0.70), while associations were found between working status at T1 and T2 and SBP change or hypertension (respectively *p* = 0.09 and *p* = 0.06 at T1, and *p* = 0.95 and *p* < 0.001 at T2).

Bivariate and multivariate linear regressions were performed to investigate the independent associations between each potential explanatory OS and T2-T1 mean SBP difference during the 5-year follow-up period (Table 2).

**Table 2:** Associations between each occupational stressors and SBP difference: 5 year-follow-up (linear regressions)

*N* = 1156	SBP difference T2-T1 Unadjusted model									SBP difference T2-T1 Adjusted model								
	Whole sample			Initial SBP < 130 mmHg			Initial SBP ≥ 130 mmHg			Whole sample			Initial SBP < 130 mmHg			Initial SBP ≥ 130 mmHg		
Occupational stressors	β.	SE	*p*	β.	SE	p	β.	SE	*p*	β.	SE	*p*	β.	SE	*p*	β.	SE	*p*
Physical risks																		
Carrying heavy loads, yes (ref: no)	0.48	1.05	0.64	2.24	1.17	**0.06**	−0.001	1.82	1.00	0.79	1.06	0.46	0.28	1.25	0.82	0.92	2.16	0.67
Intense noise, yes (ref: no)	0.41	1.20	0.73	2.84	1.43	**0.047**	0.96	1.95	0.62	0.76	1.33	0.57	−0.38	1.60	0.81	0.20	2.55	0.94
Organisational factors																		
Working at the weekend, yes (ref: no)	−1.23	1.00	0.22	−0.65	1.08	0.55	−3.03	1.81	**0.09**	−1.07	0.90	0.23	−1.10	1.06	0.30	−2.41	1.84	0.19
> 48 h /week, yes (ref: no)	0.88	1.13	0.44	3.39	1.30	**0.009**	0.52	1.90	0.78	0.31	1.18	0.80	1.21	1.41	0.39	−0.08	2.37	0.97
Rotating shifts, yes (ref: no)	0.03	1.08	0.98	0.99	1.20	0.41	−0.001	1.90	1.00	−0.57	1.10	0.60	−0.96	1.26	0.45	−0.60	2.30	0.80
Bedtime > midnight, yes (ref: no)	1.20	1.20	0.32	2.12	1.40	0.13	2.90	1.99	0.15	1.16	1.29	0.37	−0.77	1.52	0.61	3.89	2.57	0.13
Getting up < 5 AM, yes (ref: no)	0.59	1.19	0.62	2.00	1.41	0.16	2.43	1.94	0.21	1.04	1.29	0.42	−0.99	1.55	0.52	3.92	2.49	0.12
Psychosocial factors																		
Job strain (ref: low)																		
Passive work	−1.68	1.92	0.38	0.69	2.19	0.75	−4.18	3.22	0.20	−0.79	1.74	0.65	0.16	2.14	0.94	−4.51	3.29	0.17
Active work	1.88	1.17	0.106	2.29	1.29	**0.08**	0.34	2.06	0.87	2.38	1.07	**0.03**	2.33	1.27	**0.07**	1.49	2.10	0.48
High strain	2.00	2.16	0.36	4.02	2.25	**0.08**	−6.18	4.33	0.16	1.96	1.94	0.31	4.64	2.18	**0.03**	−3.94	4.32	0.36
Work under time pressure, yes (ref: no)	−0.21	1.33	0.87	2.31	1.56	0.14	−0.63	2.17	0.77	−0.75	1.55	0.63	−0.93	1.80	0.61	−3.65	3.16	0.25
Job recognition, yes (ref: no)	−1.92	1.18	0.10	−3.48	1.26	**0.006**	2.25	2.20	0.31	−1.26	1.07	0.24	−3.19	1.24	**0.01**	2.30	2.21	0.30
Income-productivity	0.28	1.38	0.84	2.81	1.64	**0.09**	0.15	2.22	0.95	−1.75	1.67	0.30	−0.99	1.91	0.61	−1.51	3.52	0.67
Employment factors																		
First job age (ref: 18–20 yo)																		
< 18	0.22	1.12	0.85	0.46	1.33	0.73	3.46	1.76	**0.05**	0.79	1.07	0.46	−1.17	1.36	0.39	3.26	1.92	**0.09**
> 20	0.93	1.11	0.40	0.19	1.19	0.87	0.60	2.02	0.77	1.17	1.21	0.34	0.83	1.39	0.55	1.22	2.54	0.63
Blue-collars (ref: white collars)	−0.67	0.94	0.47	0.41	1.06	0.70	−1.72	1.56	0.27	−0.64	1.03	0.54	0.44	1.32	0.74	−3.04	1.83	0.098

*Footnotes:* Each model was adjusted for individual characteristics evaluated at T1 (age group at eligibility, gender, BMI, smoking habits, daily alcohol intake, leisure time physical activity, diabetes, hypercholesterolemia), for treatment for hypertension at T1 and T2, employment at T1 and T2, and educational attainment, and initial BP level (for the whole sample)

Significance of *p*-value < 0.5 are set in bold

Within the whole sample, simple linear regression analyses highlighted a borderline significant link between some OS and SBP changes. After adjustment, only a significant association remained between being exposed to job strain and the T2-T1 mean SBP increase.

In people with baseline SBP < 130 mmHg, significant or borderline significant associations were observed with an increased SBP over time: positive associations with exposure to carrying heavy loads (p = 0.06), intense noise exposure (*p* = 0.047), working more than 48 h/week (*p* = 0.009), active (*p* = 0.08), high strain (p = 0.08), income linked to productivity (p = 0.09) and an inverse relationship with job recognition (*p* = 0.006). After adjustments, the links between OS and mean SBP change disappeared except for job strain and job recognition.

For participants with initial SBP ≥ 130 mmHg, a negative association with an increased difference of SBP between T2 and T1 was highlighted among participants working at the weekend (*p* < 0.10) only in an unadjusted model.

**Fig. 1:**
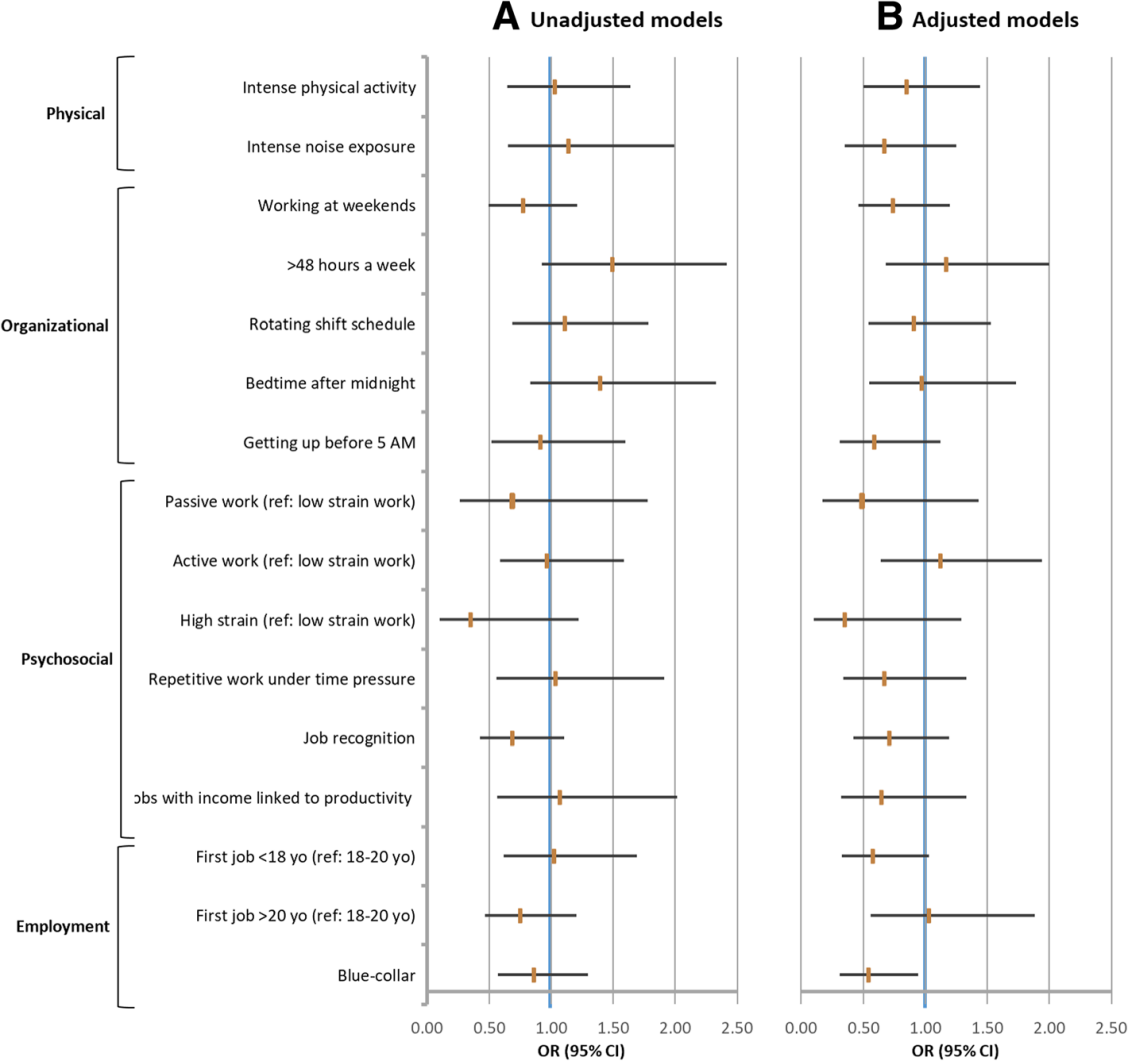
Associations between each occupational environmental constraint (considered separately) and incident cases of hypertension: 5-year follow-up. **a**: unadjusted model; **b**: adjusted model (age group at eligibility, gender, BMI, smoking habits, daily alcohol intake, leisure time physical activity, diabetes, hypercholesterolemia), employment at T1 and T2, and educational attainment. Footnotes: OR, Odds-ratio; 95% CI, 95% Confidence Interval

Before and after adjustments, no OS was significantly associated to hypertension incident cases between T2 and T1 (unadjusted and adjusted logistic regressions: Fig. 1a, b).

Backward multivariate analyses initially including all OS associated (*p* < 0.20) with SBP change or hypertension incident cases in bivariate analyses were carried out.

In the whole population, after taking into account all the related confounders (*p*-value < 0.20) and notably the initial BP level, no OS appeared to be associated with BP change.

However, for workers whose initial SBP was < 130 mmHg, being exposed to an active job strain was associated (borderline significant) with a higher risk of an SBP increase over the 5-year follow-up, while job recognition decreased this risk significantly (Table 3).

**Table 3:** Occupational stressors and SBP or incident cases of hypertension (backward multivariate analysis)

	SBP difference T2-T1									Incidence of Hypertension (model D) *N* = 775		
	Whole sample (model A) *N* = 1156			Initial SBP < 130 mmHg (model B) *N* = 673			Initial SBP ≥ 130 mmHg (model C) *N* = 483					
	β.	SE	*p*	β.	SE	*p*	β.	SE	*p*	OR	95% CI	*p*
Age group at baseline (ref: 32 years old)												
42	3.10	1.01	**0.002**	1.12	1.14	0.33	3.89	2.20	**0.08**	2.36	(1.41; 3.96)	**0.001**
52 or 62	7.02	1.25	**< 0.001**	5.13	1.61	**0.001**	5.52	2.43	**0.02**	4.97	(2.68; 9.23)	**< 0.001**
Female (ref male)	−3.02	0.98	**0.002**	−0.49	1.08	0.65	−2.61	1.99	0.19	0.53	(0.34; 0.82)	**0.005**
BMI at T1 (kg/m^2^)	0.49	0.12	**< 0.001**	0.61	0.14	**< 0.001**	–	–	–	1.18	(1.11; 1.25)	**< 0.001**
Drugs for HBP T1	4.72	1.77	**0.01**	–	–	–	8.39	3.22	**0.01**	–	–	–
Drugs for HBP T2				–	–	–	−6.19	2.73	**0.02**	–	–	–
SBP at T1	−0.45	0.03	**< 0.001**									
Smoking (ref: no)	2.17	0.97	**0.03**	–	–	–	3.98	1.98	**0.05**	1.74	(1.10; 2.76)	**0.02**
Daily alcohol intake												
Yes, without alcohol dependence	–	–	–	–	–	–	−1.46	1.92	0.45	–	–	–
Yes, with alcohol dependence	–	–	–	–	–	–	−7.75	3.38	**0.02**	–	–	–
Working at T1	–	–	–	−8.27	3.08	**0.01**	–	–	–	–	–	–
Job strain (ref: low strain work)												
Passive work	–	–	–	−0.23	2.16	0.92	–	–	–	–	–	–
Active work	–	–	–	2.24	1.26	**0.08**	–	–	–	–	–	–
High strain	–	–	–	3.43	2.24	0.13	–	–	–	–	–	–
Job recognition	–	–	–	−3.09	1.27	**0.02**	–	–	–	–	–	–

*Footnotes:* β and standard errors, odds ratio and 95% confidence intervals were calculated from linear and logistic regressions respectively

In all models the following variables were included: age group at eligibility, gender, BMI, smoking, daily alcohol intake, history of diabetes, lipid disorders, educational attainment, and employment at T1 and at T2

Added in model A: initial SBP level, HBP treatment at T1 and T2, leisure physical activity; job strain, and job recognition

Added in model B: HBP treatment at T1 and T2, leisure physical activity; carrying heavy objects, intense noise exposure, > 48 h/week, bedtime after midnight, getting up before 5 AM, job strain, work under time pressure, job recognition, and income linked to productivity

Added in model C: HBP treatment at T1 and T2, leisure physical activity, working on the weekend, bedtime after midnight, getting up before 5 AM and age at first job

Added in model D: working during the weekend, > 48 h/week, rotating shift schedule and job recognition

Significance of *p*-value < 0.5 are set in bold

None of the OS was associated with either SBP increase in the ≥ 130 mmHg group or hypertension incident cases over the five-year follow-up.

## Discussion

The VISAT study allowed us to explore the specific consequences of a broad set of OS on BP increase and on incident cases of hypertension during a 5-year follow-up. The analyses were conducted to evaluate the impact of each OS separately but also in models considering various relevant individual variables and several OS which play a potential role on SBP changes and on incidence of hypertension.

Four main messages were highlighted:

1. Some OS were associated with the increase of SBP five years later only when the initial SBP level did not exceed 130 mmHg, considered as the normal value limit of BP. Thus, in unadjusted models, carrying heavy loads, exposure to intense noise, working more than 48 h/week, exposure to atypical work schedules (working hours disturbing sleep), job strain and having a job with incomes linked to productivity tended to be associated with an increase of T2-T1 mean SBP difference, while job recognition had a protective effect.
2. However, the adjusted models revealed that most of these associations were explained by individual cardiovascular factors, except for the negative effect of high job strain and positive effect of job recognition which had an independent role.
3. The OS did not act on SBP five years later when initial mean SBP level was above 130 mmHg, and when considering incidence of hypertension as defined by the WHO (≥140/90 mmHg and/or specific treatment).
4. Because workers are exposed to several OS during the working period, for the first time, combined OS and individual determinants were considered in the same model. Only the protective role of job recognition on SBP change over 5 years (with initial SBP level < 130 mmHg) remained significant. These results suggest the potential compensatory effect between OS.

The mean SBP change over both collections is very small, evaluated at 1.5 mmHg with SD at 14.9 mmHg. It could appear of little significance but it is well demonstrated that the risk of cardiovascular mortality raises linearly with the increase of BP and a SBP decrease of 2 mmHg would lead to about 10% lower stroke mortality and about 7% lower mortality from CVD or other vascular causes in middle age [1]. Recently, the Minnesota and Zutphen Studies demonstrated that a linear increase of SBP over 10 years increases the risk of cardiovascular mortality, depending on the level of SBP at baseline [13].

Regarding occupational physical constraints, a growing interest was paid over the last years to the impact of occupational PA (e.g., carrying heavy loads) on cardiovascular events or mortality [21–23] Few studies have focused on its impact on BP with contrasted results and inconstant adjustments [9, 11]. In the present study, carrying heavy loads or being exposed to an intense PA at work tended to increase the SBP difference between T2 and T1 but not after controlling for the main cardiovascular risk factors. A relationship has been shown between intense noise exposure and SBP increase in the current study for people who had a normal initial SBP level, but it did not remain significant after adjustment for main cardiovascular risk factors in accordance with the findings from the Helsinki Heart study [11]. In contrast Van Kempen et al. earlier highlighted a higher risk of hypertension (estimated at 14%) per unit of occupational noise level [10]. However, being exposed for a long time (between 20 and 30 years) to a high intensity noise in the working environment seems necessary to observe an increased risk of hypertension, as a result of a cumulative exposure effect [24, 25]. Several pathophysiological hypotheses have been suggested such as an increase of systemic vascular resistance and also an increase of stress hormones secretion [26] . The question used in the VISAT study to assess noise exposure did not allow specifying neither the cumulative number of years of exposure, nor the exact level of noise intensity, but the definition used here was broadly used in other French surveys to define an intense noise. Based on this definition, 18% of French workers were exposed to this type of OS [27].

Though long working hours at the workplace are spread, their impact on BP remains unclear and has been little explored so far. A significant inverse association between overtime work and the prevalence of hypertension was found in some studies [28, 29]. In contrast an increased BP was observed among people exposed to long working hours depending on the occupational social categories and job tasks [30]. With a longer follow-up, a larger sample and adjustment for a large set of OS, an effect of overtime (> 48 h/week) on SBP was demonstrated among participants with normal initial SBP, mainly explained by individual cardiovascular risk factors, in the current prospective study. The review of the literature undertaken between 2000 and 2010 [8, 9] suggested a potential impact of shift work on hypertension, but due to the heterogeneity of studies (sample size, duration of follow-up and design study), this effect needed to be confirmed. The findings of the present study do not confirm any impact of shift work on BP change after adjustment for the main individual factors and other OS suspected to potentially modify BP.

Both a systematic review [12] and a meta-analysis [7] confirmed the adverse effect of job psychosocial constraints on BP with a more consistent negative effect for men compared to women. Because of the few numbers of longitudinal studies included and their design heterogeneity, a conclusion was difficult to draw, although it seems that a 4 to 5 mmHg increase of BP can be retained when people are exposed to job strain. However, few other OS were retained as potential confounding factors in these studies. Our results confirmed the small adverse effect of job strain on SBP change, which completely disappeared when all individual and other OS were included in models, while job recognition confirmed its protective effect. These findings question about the counterbalanced effects of the different OS when there are considered together. This could be an explanation to the heterogeneity of previous findings.

The strength of this study consists in the investigation of the relationships between several OS (some of them scarcely explored before) and SBP change and hypertension in a large cohort with both male and female workers. Moreover, analyses were performed with factors known to be related to BP, but also while considering the combined OS. The family history of hypertension was not available in this study and could be introduced in the next study on this topic.

Findings of the present study suggest an impact of some OS on SBP when initial SBP level was lower than 130 mmHg but none on incident cases of hypertension. Several assumptions can be formulated: firstly, the OS has an influence on SBP, but not a major one. Thus, the OS could potentiate the negative effects of the main well-known cardiovascular risk factors. Secondly, the 5-year duration of the follow-up in the VISAT study is interesting compared to those proposed in other studies, but it may still not be sufficient to perceive the effect of OS on incident cases of hypertension. Thirdly, the exposure duration of each OS could not be specified in the present study and a dose–response effect on hypertension could be suspected and could constitute a next step to explore in future studies.

Despite using the mean of three measures of blood pressure at T1 and T2 and the interest to consider the 130 mmHg threshold (limit normal BP) [13], the frequently occurring phenomenon known as regression toward the mean in these repeated data can be observed and considered as a possible cause of the observed changes, in particular when participants are categorised based on their SBP baseline measurements.

## Conclusion

In this article we perform the first assessment of the association between a large panel of occupational environmental stressors and blood pressure, when occupational environmental stressors were considered separately and in combination in a French general working population (including men and women). Occupational environmental stressors play a role on SBP mainly when initial SBP is in normal range but not on incident cases of hypertension over a 5-year follow-up. Cardiovascular risk factors explain most of these associations. The recognition of completed tasks has an independent protective role on SBP.

Highlighting the adverse effects of some organisational and psychosocial factors could be helpful in order to implement primary prevention strategies in the workplace by occupational health teams and at individual level by cardiologists and general practitioners.

## Additional files

Additional file 1: Table A characteristics of drop-outs and included participants. (DOCX 13 kb)

Additional file 2: Table B mean and SD (mmHg) of each BP measure at each examination. (DOCX 13 kb)

Additional file 3: Table C participant’s characteristics according to initial SBP level. (DOCX 21 kb)

Additional file 4: Table D occupational characteristics according to SBP difference and incident cases of Hypertension: 5 year-follow-up. (DOCX 30 kb)

## Abbreviations

BP: Blood pressure
CVD: Cardiovascular diseases
DL: Decision latitude
HBP: Hypertension
OR: Odds ratios
OS: Occupational environmental stressors
PA: Physical activity
PD: Psychological demand
SBP: Systolic blood pressure
SD: Standard deviation
