# Supplementary material for: Impact of occupational environmental stressors on blood pressure changes and on incident cases of hypertension: a 5-year follow-up from the VISAT study
Source: Environ Health. 2018 Nov 16;17:79. doi: 10.1186/s12940-018-0423-9 (PMC6240201; doi:10.1186/s12940-018-0423-9)
Supplement: Supplementary file 1 — Table A characteristics of drop-outs and included participants. (DOCX 13 kb) [file 12940_2018_423_MOESM1_ESM.docx]

Additional file 1: Characteristics of drop-outs and included participants

|  | Total N=2284 | Drop-outs or missing data for SBP or HBP (N=1,128) | Included (N=1,156) | p |
| --- | --- | --- | --- | --- |
| Age groups, years, % |  |  |  | <0.001 |
| 32 y | 28.2 | 25.1 | 31.3 |  |
| 42 y | 32.0 | 30.8 | 33.3 |  |
| 52 or 62 y | 39.7 | 44.1 | 35.4 |  |
| Male, % | 51.1 | 49.5 | 52.7 | 0.13 |
| BMI at T1 (kg/m²), mean ± SD | 25.3 ± 4.1 | 25.3 ± 4.1 | 25.3 ± 4.1 | 0.84 |
| Smoking at T1, yes, % | 27.7 | 27.1 | 28.3 | 0.57 |
| Daily alcohol intake at T1, % |  |  |  | 0.14 |
| Yes, no dependence | 23.0 | 22.6 | 23.5 |  |
| Yes, dependence | 5.9 | 6.9 | 5.0 |  |
| Leisure physical activity at T1, yes, % |  |  |  | 0.98 |
| Active or very active | 42.6 | 42.6 | 42.6 |  |
| > A-degree level, yes, % | 30.0 | 27.7 | 32.3 | 0.02 |
| Diabetes at T1, yes, % | 3.2 | 4.3 | 2.2 | 0.01 |
| Hypercholesterolemia at T1, yes, % | 18.1 | 21.3 | 15.1 | <0.001 |
| SBP at T1, mean ± SD | 127.6 ± 18.8 | 128.2 ± 19.0 | 127.0 ± 18.6 | 0.17 |
| DBP at T1, mean ± SD | 79.8 ± 12.8 | 79.9 ± 14.0 | 79.8 ± 11.6 | 0.84 |
| Drugs for HBP T1, yes, % | 11.7 | 13.3 | 10.1 | 0.02 |
| Working at T1, yes, % | 81.9 | 80.2 | 83.5 | 0.04 |

Categorical variables were assessed using Chi-squared, continuous variables were assessed using Student’s t test or Mann-Whitney test.
