# Supplementary material for: Impact of occupational environmental stressors on blood pressure changes and on incident cases of hypertension: a 5-year follow-up from the VISAT study
Source: Environ Health. 2018 Nov 16;17:79. doi: 10.1186/s12940-018-0423-9 (PMC6240201; doi:10.1186/s12940-018-0423-9)
Supplement: Supplementary file 2 — Table B mean and SD (mmHg) of each BP measure at each examination. (DOCX 13 kb) [file 12940_2018_423_MOESM2_ESM.docx]

Additional file 2: Mean and SD (mmHg) of each BP measure at each examination

|  | SBP | | | | | DBP | | | | |
| --- | --- | --- | --- | --- | --- | --- | --- | --- | --- | --- |
|  | T1 |  | T2 |  | p* | T1 |  | T2 |  | p* |
|  | Mean | SD | Mean | SD |  | Mean | SD | Mean | SD |  |
| 1st measure | 128.5 | 19.9 | 130.1 | 20.0 |  | 80.5 | 12.8 | 79.7 | 12.1 |  |
| 2nd measure | 126.5 | 19.5 | 128.3 | 19.2 |  | 79.6 | 12.6 | 78.8 | 12.1 |  |
| 3rd measure | 126.2 | 19.8 | 127.3 | 19.3 |  | 79.2 | 13.3 | 78.5 | 11.9 |  |
| Mean, SD | 127.1 | 18.6 | 128.6 | 18.7 | 0.001 | 79.8 | 11.6 | 79.0 | 11.2 | 0.011 |

*p-value for Student’s t-test
