# Supplementary material for: Impact of occupational environmental stressors on blood pressure changes and on incident cases of hypertension: a 5-year follow-up from the VISAT study
Source: Environ Health. 2018 Nov 16;17:79. doi: 10.1186/s12940-018-0423-9 (PMC6240201; doi:10.1186/s12940-018-0423-9)
Supplement: Supplementary file 3 — Table C participant’s characteristics according to initial SBP level. (DOCX 21 kb) [file 12940_2018_423_MOESM3_ESM.docx]

Additional file 3: Participants characteristics according to initial SBP level

|  | Initial SBP<130 mmHg (N=673) | | Initial SBP≥130 mmHg (N=483) | | p |
| --- | --- | --- | --- | --- | --- |
|  | No. | % | No. | % |  |
| **Individual characteristics** |  |  |  |  |  |
| Age groups, years, % |  |  |  |  |  |
| 32 y | 269 | 40.0 | 93 | 19.3 | <0.001 |
| 42 y | 247 | 36.7 | 138 | 28.6 |  |
| 52 or 62 y | 157 | 23.3 | 252 | 52.2 |  |
| Male, % | 258 | 38.3 | 351 | 72.7 | <0.001 |
| BMI at T1 (kg/m²), mean ± SD | 232 | 35.1 | 303 | 64.2 | <0.001 |
| Smoking at T1, yes, % | 194 | 29.8 | 116 | 26.0 | 0.16 |
| Daily alcohol intake at T1, % |  |  |  |  | <0.001 |
| Yes, no dependence | 101 | 15.2 | 168 | 35.1 |  |
| Yes, dependence | 16 | 2.4 | 41 | 8.6 |  |
| Leisure physical activity at T1, yes, % |  |  |  |  | 0.51 |
| Active or very active | 286 | 43.5 | 195 | 41.5 |  |
| > A-degree level, yes, % | 254 | 37.8 | 119 | 24.7 | <0.001 |
| Diabetes at T1, yes, % | 10 | 1.5 | 16 | 3.3 | 0.04 |
| Hypercholesterolemia at T1, yes, % | 82 | 12.2 | 92 | 19.1 | 0.001 |
| **Occupational stressors** |  |  |  |  |  |
| **Physical risks** |  |  |  |  |  |
| Carrying heavy loads, yes | 179 | 28.1 | 139 | 35.6 | 0.01 |
| Intense noise, yes | 101 | 15.8 | 110 | 28.2 | <0.001 |
| **Organisational factors** |  |  |  |  |  |
| Working at the weekend, yes | 252 | 39.5 | 143 | 36.7 | 0.37 |
| >48hours /week, yes | 128 | 20.1 | 122 | 31.4 | <0.001 |
| Rotating shifts, yes | 159 | 25.0 | 114 | 29.5 | 0.12 |
| Bedtime > midnight, yes | 104 | 16.3 | 98 | 25.2 | 0.001 |
| Getting up < 5 AM, yes | 102 | 16.0 | 111 | 28.6 | <0.001 |
| Job strain |  |  |  |  | 0.14 |
| low strain | 147 | 23.3 | 103 | 26.6 |  |
| passive work | 48 | 7.6 | 39 | 10.1 |  |
| active work | 386 | 61.3 | 225 | 58.1 |  |
| high strain | 49 | 7.8 | 20 | 5.2 |  |
| Work under time pressure, yes | 83 | 13.0 | 77 | 19.8 | 0.004 |
| Job recognition, yes | 496 | 77.9 | 307 | 79.3 | 0.58 |
| Income-productivity, yes | 74 | 11.7 | 74 | 19.0 | 0.001 |
| **Employment factors** |  |  |  |  |  |
| First job age |  |  |  |  | <0.001 |
| <18 | 162 | 24.1 | 199 | 41.2 |  |
| 18-20 | 272 | 40.4 | 162 | 33.5 |  |
| >20 | 239 | 35.5 | 122 | 25.3 |  |
| Blue-collars | 377 | 57.3 | 279 | 59.4 | 0.49 |

p. p-value for Chi-squared test
