# Supplementary material for: Impact of occupational environmental stressors on blood pressure changes and on incident cases of hypertension: a 5-year follow-up from the VISAT study
Source: Environ Health. 2018 Nov 16;17:79. doi: 10.1186/s12940-018-0423-9 (PMC6240201; doi:10.1186/s12940-018-0423-9)
Supplement: Supplementary file 4 — Table D occupational characteristics according to SBP difference and incident cases of Hypertension: 5 year-follow-up. (DOCX 30 kb) [file 12940_2018_423_MOESM4_ESM.docx]

Additional file 4: Occupational characteristics according to SBP difference and incident cases of Hypertension: 5 year-follow-up

|  | SBP Difference T2-T1 | | | | | | | | |  | Incident cases of Hypertension at T2 Participants without HBP at T1 | | | |
| --- | --- | --- | --- | --- | --- | --- | --- | --- | --- | --- | --- | --- | --- | --- |
|  | Whole sample (N=1,156) | | |  | Initial SBP <130 mmHg (N=673) | |  | Initial SBP≥130 mmHg (N=483) | |  | HBP incidence 1000 person-year  (N=775) | No HBP (N=641) | HBP (N=134) | p |
|  | % | mean ± SD | p |  | mean ± SD | p |  | mean ± SD | p |  |  | % | % |  |
| **Physical risks** | | | |  |  |  |  |  |  |  |  |  |  |  |
| Carrying heavy loads | | | |  |  |  |  |  |  |  |  |  |  |  |
| No | 69.1 | 1.6 ± 14.5 | 0.33 |  | 4.8 ± 12.8 | **0.02** |  | -4.1 ± 15.6 | 0.75 |  | 33.3 | 71.5 | 70.2 | 0.76 |
| Yes | 30.9 | 2.6 ± 15.6 |  |  | 7.4 ± 12.7 |  |  | -3.5 ± 16.9 |  |  | 35.6 | 28.5 | 29.8 |  |
| Intense noise | | | |  |  |  |  |  |  |  |  |  |  |  |
| No | 79.5 | 1.9 ± 14.1 | 0.48 |  | 5 ± 12.6 | **0.06** |  | -4.2 ± 14.9 | 0.39 |  | 32.5 | 84.6 | 80.6 | 0.28 |
| Yes | 20.5 | 2.2 ± 17.3 |  |  | 7.8 ± 13.6 |  |  | -3 ± 18.7 |  |  | 41.6 | 15.4 | 19.4 |  |
| **Organisational factors** | | | |  |  |  |  |  |  |  |  |  |  |  |
| Working at weekends | | | |  |  |  |  |  |  |  |  |  |  |  |
| No | 61.6 | 2.3 ± 14.6 | 0.28 |  | 5.5 ± 13.1 | 0.96 |  | -2.6 ± 15.5 | **0.046** |  | 36.5 | 60.7 | 66.9 | 0.19 |
| Yes | 38.4 | 1.3 ± 15.1 |  |  | 5.4 ± 12.4 |  |  | -6 ± 16.8 |  |  | 29.8 | 39.3 | 33.1 |  |
| >48 hours/week | | | |  |  |  |  |  |  |  |  |  |  |  |
| No | 75.6 | 1.9 ± 14.2 | 0.78 |  | 4.9 ± 12.6 | **0.02** |  | -3.8 ± 15.3 | 0.98 |  | 31.3 | 80.4 | 72.6 | **0.05** |
| Yes | 24.4 | 2.2 ± 16.6 |  |  | 8 ± 13.2 |  |  | -3.8 ± 17.6 |  |  | 44.4 | 19.6 | 27.4 |  |
| Rotating shift |  |  |  |  |  |  |  |  |  |  |  |  |  |  |
| No | 73.3 | 1.9 ± 14.5 | 0.98 |  | 5.1 ± 12.8 | 0.27 |  | -3.6 ± 15.8 | 0.68 |  | 33.0 | 75.2 | 74.0 | 0.78 |
| Yes | 26.7 | 1.9 ± 15.5 |  |  | 6.4 ± 12.8 |  |  | -4.4 ± 16.7 |  |  | 36.1 | 24.8 | 26.0 |  |
| Bedtime > midnight | | | |  |  |  |  |  |  |  |  |  |  |  |
| No | 80.3 | 1.7 ± 14.2 | 0.35 |  | 5.2 ± 12.5 | 0.30 |  | -4.7 ± 14.7 | **0.11** |  | 32.2 | 83.6 | 79.0 | 0.22 |
| Yes | 19.7 | 3 ± 17.3 |  |  | 7.2 ± 13.9 |  |  | -1.5 ± 19.4 |  |  | 42.8 | 16.4 | 21.0 |  |
| Getting up < 5 AM | | | |  |  |  |  |  |  |  |  |  |  |  |
| No | 79.2 | 1.8 ± 14.4 | 0.56 |  | 5.1 ± 12.8 | 0.15 |  | -4.7 ± 15 | 0.12 |  | 33.7 | 82.9 | 83.1 | 0.96 |
| Yes | 20.8 | 2.5 ± 16.4 |  |  | 7.1 ± 12.3 |  |  | -1.8 ± 18.4 |  |  | 35.2 | 17.1 | 16.9 |  |
| **Psychosocial factors** | | | |  |  |  |  |  |  |  |  |  |  |  |
| Job strain |  |  |  |  |  |  |  |  |  |  |  |  |  | 0.47 |
| low strain | 24.6 | 0.3 ± 15.2 | **0.07** |  | 3.6 ± 13.4 | **0.10** |  | -4.5 ± 16.5 | 0.40 |  | 36.6 | 23.0 | 25.2 |  |
| passive work | 8.6 | -0.3 ± 15.5 |  |  | 4 ± 11.4 |  |  | -5.5 ± 18.2 |  |  | 30.7 | 7.7 | 6.5 |  |
| active work | 60.1 | 2.7 ± 14.5 |  |  | 5.9 ± 12.9 |  |  | -2.9 ± 15.4 |  |  | 35.3 | 61.5 | 64.2 |  |
| high strain | 6.8 | 3.4 ± 15.3 |  |  | 8.2 ± 11.1 |  |  | -8.4 ± 17.7 |  |  | 19.4 | 7.8 | 4.1 |  |
| Time pressure |  |  |  |  |  |  |  |  |  |  |  |  |  |  |
| No | 84.4 | 1.9 ± 14.3 | 0.78 |  | 5.2 ± 12.6 | 0.23 |  | -3.8 ± 15.5 | 0.69 |  | 33.6 | 87.0 | 86.3 | 0.84 |
| Yes | 15.6 | 1.9 ± 17.3 |  |  | 7.5 ± 14.3 |  |  | -4.1 ± 18.4 |  |  | 36.8 | 13.0 | 13.7 |  |
| Job recognition |  |  |  |  |  |  |  |  |  |  |  |  |  |  |
| No | 21.6 | 3.7 ± 15.5 | **0.04** |  | 8.4 ± 13.3 | **0.003** |  | -4.4 ± 15.8 | 0.80 |  | 42.3 | 21.1 | 27.4 | 0.12 |
| Yes | 78.4 | 1.4 ± 14.6 |  |  | 4.7 ± 12.6 |  |  | -3.9 ± 16 |  |  | 31.7 | 78.9 | 72.6 |  |
| Income-productivity | | | |  |  |  |  |  |  |  |  |  |  |  |
| No | 85.5 | 2 ± 14.5 | 0.75 |  | 5.2 ± 12.7 | 0.15 |  | -3.8 ± 15.7 | 0.77 |  | 33.7 | 88.1 | 87.8 | 0.93 |
| Yes | 14.5 | 1.6 ± 16.9 |  |  | 7.5 ± 13.8 |  |  | -4.4 ± 17.6 |  |  | 35.8 | 11.9 | 12.2 |  |
| **Employment factors** | | | |  |  |  |  |  |  |  |  |  |  |  |
| Age of First job |  |  |  |  |  |  |  |  |  |  |  |  |  | 0.72 |
| <18 y | 31.2 | 1.4 ± 15.4 | 0.58 |  | 6.1 ± 13.3 | 0.81 |  | -2.5 ± 16 | 0.12 |  | 38.0 | 22.9 | 25.4 |  |
| 18 to 20 y | 37.5 | 1.1 ± 15 |  |  | 5.3 ± 12.8 |  |  | -5.9 ± 15.9 |  |  | 35.4 | 40.9 | 41.8 |  |
| >20 y | 31.2 | 2.2 ± 14.2 |  |  | 5.5 ± 12.6 |  |  | -4.3 ± 15.1 |  |  | 31.9 | 36.2 | 32.8 |  |
| Occupational status | | | |  |  |  |  |  |  |  |  |  |  |  |
| Blue-collar | 58.2 | 1.5 ± 15.1 | 0.77 |  | 5.7 ± 12.7 | 0.58 |  | -4.2 ± 16.1 | 0.51 |  | 31.9 | 57.5 | 51.9 | 0.24 |
| White-collar | 41.8 | 1.7 ± 14.4 |  |  | 5.2 ± 13.1 |  |  | -3.3 ± 14.7 |  |  | 38.3 | 42.5 | 48.1 |  |
| Working at T1 |  |  |  |  |  |  |  |  |  |  |  |  |  |  |
| No | 16.5 | -0.2 ± 17.5 | **0.09** |  | 10.7 ± 15.1 | **0.01** |  | -4.7 ± 16.5 | 0.90 |  | 51.6 | 7.8 | 12.8 | **0.06** |
| Yes | 83.5 | 1.9 ± 14.3 |  |  | 5.1 ± 12.5 |  |  | -3.9 ± 15.5 |  |  | 32.9 | 92.2 | 87.2 |  |
| Working at T2 |  |  |  |  |  |  |  |  |  |  |  |  |  |  |
| No | 32 | 1.3 ± 17.1 | 0.95 |  | 9.7 ± 14.8 | **<0,001** |  | -3.7 ± 16.4 | 0.32 |  | 58.1 | 17.6 | 34.3 | **<0.001** |
| Yes | 68 | 1.6 ± 13.8 |  |  | 4.5 ± 12.1 |  |  | -4.4 ± 15.2 |  |  | 28.7 | 82.4 | 65.7 |  |
| Job change (T1 - T2) | | | |  |  |  |  |  |  |  |  |  |  |  |
| No | 90.8 | 1.9 ± 14.3 | 0.57 |  | 5.2 ± 12.5 | 0.43 |  | -3.9 ± 15.4 | 0.28 |  | 32.7 | 89.8 | 91.0 | 0.70 |
| Yes | 9.2 | 0.9 ± 15.3 |  |  | 3.9 ± 13.9 |  |  | -7.6 ± 16.1 |  |  | 28.7 | 10.2 | 9.0 |  |

Footnotes: percentages or means (SD). Categorical variables were assessed using Chi-squared, continuous variables were assessed using Student’s t test or Mann-Whitney test.
